# A Multidisciplinary Approach for the Personalised Non-Operative Management of Elderly and Frail Rectal Cancer Patients Unable to Undergo TME Surgery

**DOI:** 10.3390/cancers14102368

**Published:** 2022-05-11

**Authors:** Stijn H. J. Ketelaers, Anne Jacobs, An-Sofie E. Verrijssen, Jeltsje S. Cnossen, Irene E. G. van Hellemond, Geert-Jan M. Creemers, Ramon-Michel Schreuder, Harm J. Scholten, Jip L. Tolenaar, Johanne G. Bloemen, Harm J. T. Rutten, Jacobus W. A. Burger

**Affiliations:** 1 Department of Surgery, Catharina Hospital, P.O. Box 1350, 5602 ZA Eindhoven, The Netherlands; jip.tolenaar@catharinaziekenhuis.nl (J.L.T.); johanne.bloemen@catharinaziekenhuis.nl (J.G.B.); harm.rutten@catharinaziekenhuis.nl (H.J.T.R.); pim.burger@catharinaziekenhuis.nl (J.W.A.B.); 2 Department of Gerontology and Geriatrics, Catharina Hospital, P.O. Box 1350, 5602 ZA Eindhoven, The Netherlands; anne.jacobs@catharinaziekenhuis.nl; 3 Department of Radiation Oncology, Catharina Hospital, P.O. Box 1350, 5602 ZA Eindhoven, The Netherlands; an-sofie.verrijssen@catharinaziekenhuis.nl (A.-S.E.V.); jeltsje.cnossen@catharinaziekenhuis.nl (J.S.C.); 4 Department of Medical Oncology, Catharina Hospital, P.O. Box 1350, 5602 ZA Eindhoven, The Netherlands; irene.v.hellemond@catharinaziekenhuis.nl (I.E.G.v.H.); geert-jan.creemers@catharinaziekenhuis.nl (G.-J.M.C.); 5 Department of Gastroenterology, Catharina Hospital, P.O. Box 1350, 5602 ZA Eindhoven, The Netherlands; ramonmichel.schreuder@catharinaziekenhuis.nl; 6 Department of Anaesthesiology, Catharina Hospital, P.O. Box 1350, 5602 ZA Eindhoven, The Netherlands; harm.scholten@catharinaziekenhuis.nl; 7 GROW, School for Oncology and Reproduction, Maastricht University, P.O. Box 616, 6200 MD Maastricht, The Netherlands

**Keywords:** rectal cancer, non operative management, elderly patients, frailty, multidisciplinary, personalised care, patient-centred approach

## Abstract

**Simple Summary:**

Total mesorectal excision is the cornerstone for rectal cancer curation. However, elderly and frail patients may not be able to undergo a surgical procedure. These patients often receive no treatment at all and are at risk for developing debilitating symptoms that impair quality of life. Recent developments in the non-operative management of rectal cancer have increased the possibilities to provide patients with an alternative treatment if surgery is not possible, in an effort to avoid the onset of debilitating symptoms, improve quality of life, and prolong survival. The heterogeneity within the elderly and frail population requires a patient-centred approach to optimise treatment. The aim of this narrative review was to discuss a multidisciplinary and patient-centred treatment approach for the personalised non-operative management of elderly and frail rectal cancer patients. The narrative review also provides a practical suggestion of a successfully implemented multidisciplinary clinical care pathway, based on a literature review.

**Abstract:**

Despite it being the optimal curative approach, elderly and frail rectal cancer patients may not be able to undergo a total mesorectal excision. Frequently, no treatment is offered at all and the natural course of the disease is allowed to unfold. These patients are at risk for developing debilitating symptoms that impair quality of life and require palliative treatment. Recent advancements in non-operative treatment modalities have enhanced the toolbox of alternative treatment strategies in patients unable to undergo surgery. Therefore, a proposed strategy is to aim for the maximal non-operative treatment, in an effort to avoid the onset of debilitating symptoms, improve quality of life, and prolong survival. The complexity of treating elderly and frail patients requires a patient-centred approach to personalise treatment. The main challenge is to optimise the balance between local control of disease, patient preferences, and the burden of treatment. A comprehensive geriatric assessment is a crucial element within the multidisciplinary dialogue. Since limited knowledge is available on the optimal non-operative treatment strategy, these patients should be treated by dedicated multidisciplinary rectal cancer experts with special interest in the elderly and frail. The aim of this narrative review was to discuss a multidisciplinary patient-centred treatment approach and provide a practical suggestion of a successfully implemented clinical care pathway.

## 1. Introduction

Although total mesorectal excision (TME) surgery is the optimal approach for curation, elderly and frail rectal cancer patients may not always be able to undergo a surgical procedure [1,2,3]. In these patients, decision making is challenging, and no standardised treatment regimen or guideline is available [4,5,6]. Frequently, patients receive no treatment at all and doctors and patients wait out the natural course of the disease [1,7,8]. This often results in tumour progression and the onset of debilitating symptoms that impair quality of life. Palliative treatment may then be offered to alleviate symptoms, if possible [7,9]. 

However, improvements in chemotherapeutic and radiotherapeutic treatment modalities provide alternative non-operative treatment strategies for patients who are unable to undergo TME surgery [10,11]. These strategies may provide long-term local control of the primary tumour and avoid the early-onset of debilitating symptoms, improve quality of life, and prolong survival. In some patients, curation might even be possible.

Various evidence-based and expert-based recommendations exist on how elderly and frail rectal cancer patients should be treated surgically. However, the optimal treatment approach for patients who are unable to undergo TME surgery is still unknown. The patient complexity, as well as the risk for undertreatment or overtreatment require a patient-centred approach to propose the most optimal treatment strategy, considering the patient’s level of frailty, personal preferences, and treatment goals. 

The aim of this narrative review was to discuss a multidisciplinary patient-centred approach for the personalised non-operative management of elderly and frail rectal cancer patients unable to undergo TME surgery.

### 1.1. Current Treatment of Elderly and Frail Rectal Cancer Patients

Epidemiological data show that over 50% of rectal cancer patients are older than 70 years. Due to an improved life expectancy, this proportion of elderly patients will probably increase over the coming years [12]. The elderly population is characterised by a wide variety in health status, ranging from vital and fit to frail and unable to undergo even minor surgical procedures [4,5,6]. This heterogeneity results in a difficult balance between oncological outcomes, the burden of treatment, and functional outcomes.

#### 1.1.1. Considerations on the Surgical Treatment of Elderly Rectal Cancer Patients

TME surgery is generally accepted as the best curative treatment for rectal cancer [13]. While older studies reported high rates of postoperative morbidity and mortality in the elderly, the outcomes have improved significantly over recent years [14,15]. In a Dutch retrospective cohort of 2018 patients, the postoperative mortality of elderly patients (≥75 years) improved from 8.8% between 2006–2012 to 1.7% between 2013–2017, whereas the 1-year relative survival rates were no longer different between elderly and younger patients [15]. Similar improvements have been described by population-based data from the Netherlands Cancer Registry (NCR), which reported an improvement of the 1-year relative survival in the elderly (≥75 years) rectal cancer patient from 86.1% between 2005–2006 to 97.2% between 2015–2016 [14]. These results support the paradigm shift that patients should not be withheld surgery based on chronological age alone [14,15]. 

Particularly in the treatment of elderly and frail patients, the concept of personalised care is essential. Elderly and frail patients often consider that maintaining independence, quality of life, and functional outcomes are at least as important as oncological outcomes and survival [16,17,18]. These aspects should be discussed and incorporated in the decision-making process. Although the survival of elderly patients has improved significantly, clinicians should consider that the overall one-year mortality is still 10–15% [14,15]. In the most frail, the risks are even higher and may outweigh the benefits, with 2-year mortality rates above 40% [19]. TME surgery may also result in undesirable functional outcomes that impair quality of life. Various cohorts have reported severe functional bowel complaints (e.g., faecal incontinence, urgency) in 30–40% of patients [20,21]. Urinary dysfunction (e.g., incontinence, urgency) is reported by 30–60% of patients, while more than half of patients reported sexual dysfunction after treatment [22,23,24,25]. A Scottish population-based study showed that 12% of patients older than 80 years did not return to their preoperative living situation after surgery, while other studies showed that a significant part of the elderly experienced a deterioration in their functional status [26,27,28,29,30].

#### 1.1.2. Epidemiology of the Non-Surgical Treatment of the Elderly and Frail

Population-based data showed that 6–30% of patients of all ages with curable, stage I-III rectal cancer will not undergo surgery [2]. According to literature, there are several reasons why patients do not undergo TME surgery. Age is still considered the primary reason [3,17]. While approximately 30% of the patients aged 70 years or older did not undergo surgery, this percentage rose to more than 60% in those older than 80 years [1,4]. Patients with multiple or severe comorbidities also underwent less surgery [1,8]. While population-based studies have shown that most comorbidities are not predictive for poor outcomes, certain comorbidities (e.g., chronic cardiopulmonary disease, liver cirrhosis) severely increase the risk for treatment-induced morbidity and mortality or impair toleration for anaesthesia [31,32,33]. Advanced disease stages also resulted in higher non-resection rates, especially in the elderly [1,2,8]. Lastly, some patients refuse TME surgery despite being fit for reasons varying from personal convictions and preferences to fear of consequences (i.e., poor functional outcome or an ostomy).

#### 1.1.3. The Fate of Elderly and Frail Rectal Cancer Patients Refrained from Treatment

Although the number of patients treated with alternative modalities has increased over the years, data from the NCR still show that 30.4% of the older patients (≥70 years) who did not undergo surgery received no treatment at all [1]. This percentage increased to 37.1% and 40.9%, respectively, in those older than 80 years and those with multimorbidity (≥2 comorbidities) [1]. The 3-year overall and relative survival rates of these patients were 9% and 10%, respectively [1]. Although scarce, a few other studies reported on untreated rectal cancer patients. A retrospective study by Bethune et al. investigated the outcomes of 35 patients (mean age 87 years) and reported a mean overall survival of 18 months [7]. Another study among 79 patients (mean age 79.4 years) reported a median overall survival of 10.7 months and 2-year mortality rates of 76% [8]. Although selection bias might have occurred in these studies, not offering patients any treatment at all seems to be associated with a very poor survival [19,34]. 

Apart from poor survival rates, patients with untreated rectal cancer often develop severe symptoms that affect their lives significantly, which was also observed in the cohort of Bethune et al. [7]. Patients may present themselves with various symptoms related to tumour progression. In a group of 180 patients with incurable disease, the most commonly observed symptoms were bowel obstruction and rectal bleeding [35]. Approximately 10–25% of patients with stage IV disease presented with symptoms related to bowel obstruction [9,35,36,37]. If bowel obstruction results in colonic perforation, emergency surgery is usually required, which is associated with increased mortality in the elderly and frail and should be avoided [38]. Rectal tumour perforation may result in localised problems, such as pelvic abscesses, fistulae or pelvic pain, often requiring drainage [36]. Rectal bleeding and anaemia are also frequently observed, particularly in patients using anticoagulants [36]. An earlier study reported rectal bleeding in 24% of patients with incurable colorectal cancer, while an additional 12% suffered from anaemia [35]. In two retrospective cohorts, 37–43% of untreated patients needed at least one blood transfusion during follow-up [7,8].

### 1.2. The Need for a Personalised Non-Operative Treatment Approach

Elderly and frail rectal cancer patients unable to undergo TME surgery are at risk for undertreatment, which results in poor outcomes. However, progress has been made in non-operative treatment modalities [5,10,11]. Studies have explored the use of systemic chemotherapy, external beam radiotherapy (EBRT), endoluminal radiotherapy, and local excision with promising outcomes. The performed studies reported local control rates up to 60–90% and 1-year, 2-year, and 3-year overall survival rates of 82–100%, 63–88% and 27–82%, respectively [39,40,41,42,43]. 

The promising developments in the non-operative management of rectal cancer patients in general raises the question of whether these options should be considered in patients for whom surgery is not possible. It seems logical that maximal treatment effectiveness can be achieved when the available modalities are optimally allocated in each individual patient. This may result in improved local control of the primary tumour, thus aiming to prevent the onset of debilitating symptoms, improve quality of life, and prolong survival. Due to the heterogeneity in the elderly and frail population, a patient-centred approach, in which the patient is comprehensively evaluated by dedicated multidisciplinary experts, is required to optimise and personalise treatment. 

In this narrative review, three main topics to discuss a patient-centred approach for elderly and frail rectal cancer patients unable to undergo TME surgery will be addressed: (i) the multidisciplinary patient approach; (ii) the non-operative treatment options; (iii) the response evaluation and follow-up. Based on the literature review in the main topics, we provided a practical suggestion of a successfully implemented multidisciplinary clinical care pathway and a prospective observational cohort study that has been initiated by the authors of the study.

## 2. The Multidisciplinary Patient Approach

### 2.1. Treatment Outcomes in the Elderly and Frail

While the treatment outcomes of fit patients are generally more focused on the oncological results, the elderly and frail frequently prioritise maintaining quality of life and functional recovery [18]. Although curation might be the best oncological outcome, this is often not the main priority for the elderly and frail who are unable to undergo TME surgery. Setting the right expectations is important, since the burden of treatment and the expected impact on quality of life and functional recovery often determine the patient’s preferences [44].

As mentioned by Saur and Montroni et al., functional recovery can be divided in organ-specific functional recovery and the individual ability to regain or maintain independence [45]. Bowel, bladder, and sexual dysfunction are frequently observed after rectal cancer treatment and can severely impair quality of life. Considering the impact of the available treatment modalities on these organ functions is essential and should be discussed with the patient. Several scoring tools are available to evaluate pelvic organ functions, such as the Low Anterior Resection Syndrome (LARS) score, the International Prostate Symptoms Score (IPSS), and the International Index of Erectile Function (IIEF) [46,47,48,49,50,51]. Implementing these questionnaires at baseline, during treatment, and during follow-up may help to personalise the decision-making process, set the right expectations, and/or initiate treatment when symptoms arise.

Elderly patients value functional independence as one of the most important factors related to their well-being [52]. The loss of independence is considered as a detrimental burden of treatment [52]. Several studies have reported that 24–60% of elderly patients experienced a decline in their level of independence after treatment [27,28,29,30]. Clinicians should, consequently, prioritise the prevention of functional decline over obtaining curation when treating these patients. Moreover, preventing hospitalisation and institutionalisation, maintaining physical and cognitive functioning, and minimising the burden of disease and treatment for the patient and their relatives are also important parameters to consider. The geriatric frailty assessment can help to identify health domains at risk for deterioration and is considered a crucial element to personalise treatment [53]. 

### 2.2. Geriatric Assessment

Frailty is defined as a state of diminished physiological reserve capacity across multiple organ systems, resulting in a reduced capacity to compensate for stressors [54]. Frailty is a strong predictor for treatment-induced toxicity, reduced tolerance, loss of quality of life, and increased mortality [55,56]. Many factors contribute to frailty, including age and comorbidities [56]. Population-based data reported that 58.3–70.6% of patients aged 70–74 years suffer from multimorbidity (≥2 comorbidities), increasing to more than 80% in patients above 85 years [57]. While age and comorbidities contribute to frailty, they do not equal frailty [58]. Many elderly patients are not frail and can be treated safely by standard approaches, whereas only a few comorbidities contribute to poor outcomes [58,59]. Therefore, distinguishing the frail from the fit is crucial to optimise treatment [60].

#### Comprehensive Geriatric Assessment (CGA)

The CGA consists of a multidimensional evaluation of the patient’s health status [54,61]. A CGA can identify health problems and vulnerable areas that increase the risk of frailty, functional decline, toxicity, and mortality [61]. A systemic review among 35 studies by Hamaker et al. described that a CGA resulted in a changed treatment plan in 28% of elderly cancer patients [62]. A CGA was associated with increased treatment compliance and a reduced risk for toxicity [62]. A Cochrane review showed that a CGA resulted in improved decision-making and a diminished rate of institutionalisation after treatment [63]. A CGA can also identify health areas in need for improvement, leading to targeted interventions. 

Several domains that contribute to the onset and progression of frailty are evaluated in a CGA (Table 1) [12]. Multiple validated scoring tools are available to assess these health domains [64]. The presence of geriatric risk factors and syndromes (e.g., risk to fall, the risk of delirium), the living situation, the level of social support and the availability of a caregiver are also part of the CGA. Moreover, the exploration of the patient’s preferences and treatment goals (e.g., maintaining independence, reducing symptoms, maximising quality of life, prolonging survival, etc.) is considered crucial. By combining all of these outcomes, the benefits and risks of each treatment modality can be analysed within the multidisciplinary team.

### 2.3. Multidisciplinary Evaluation

The multidisciplinary evaluation has an important role in the treatment of rectal cancer, especially in the elderly and frail [17,82]. There are no accurate guidelines available on the non-operative management of elderly and frail rectal cancer patients and most evidence is based on data from clinical trials that excluded the elderly and frail [6]. Clinical consensus within a dedicated multidisciplinary team with expertise on the non-operative management of elderly and frail rectal cancer patients may most likely provide the optimal treatment advice [12,17,83]. 

In a patient-centred approach, the patient should be considered as the core of the decision-making process and should be involved actively. Informing patients about the benefits and risks of the available treatment options is an important element for shared-decision making [84]. An individual assessment by each member of the multidisciplinary team might be beneficial to improve the multidisciplinary discussion [84]. The geriatrician is an indispensable member of the multidisciplinary team and the CGA should have a central role within the multidisciplinary dialogue [83,85,86]. From the start of treatment until the end of follow-up, the multidisciplinary team should consider the patient’s health status, treatment goals, and preferences as central elements to personalise treatment [12].

### 2.4. Prehabilitation

Prehabilitation refers to the optimisation of the patient’s health status to prevent a future decline [87]. The compliance to and the response of prehabilitation may also contribute to better patient selection and improved decision-making. Prehabilitation often aims at improving the general health status, but the CGA can optimise prehabilitation by identifying specific areas of impairment. A systematic review showed that a CGA resulted in targeted interventions in 72% of patients [62]. A randomised study showed that a CGA with subsequent targeted interventions can effectively reduce frailty [88]. Moreover, a study among 106 colorectal cancer patients showed that particularly the frail had the largest benefit from prehabilitation [89]. Earlier studies mainly investigated prehabilitation in patients scheduled for surgery, but it is conceivable that patients scheduled for non-operative treatment also benefit from prehabilitation [90]. While the effects on the toxicity and compliance of non-operative treatment modalities are unexplored, increasing evidence shows favourable health benefits of prehabilitation programmes during chemotherapy and radiotherapy [91,92]. The improved health status achieved by prehabilitation is associated with an improved quality of life and may increase the probability for an escalation of treatment or even TME surgery [87].

## 3. Non-Operative Treatment Options

The non-operative management of rectal cancer in elderly and frail patients unable to undergo TME surgery should not be considered the same as palliative treatment. In palliative treatment, the natural course of the disease is often awaited and symptoms are treated when they arise, whereas the non-operative management is a more active approach with clear treatment goals to obtain local control of the primary tumour and prevent the onset of symptoms.

The advancements in chemotherapeutic and radiotherapeutic treatment modalities over the recent years have improved tumour responses [10,11,93,94]. Radiotherapy-based treatment strategies may result in adequate local control of the primary tumour. In fact, some patients can even be cured without the need for surgery. This has been supported by data from the International Watch and Wait Database. They reported 5-year overall and cancer-specific survival rates of 85% and 94%, respectively, among 880 patients with a clinical complete response [95]. A recent study by Haak et al. investigated the effectiveness of a watch-and-wait strategy among 43 elderly patients [96]. After a minimal follow-up of 2 years, the complete response was sustained in 88%, while the 3-year overall survival was 97% [96].

The beneficial outcomes have led to increased interest in the non-operative management of rectal cancer patients, which is especially relevant for elderly and frail patients who are not able to undergo TME surgery [5,6]. While curation would be the best possible outcome, the treatment of these patients mostly aims at achieving local control of the primary tumour. Improved tumour responses can be obtained by increased radiotherapeutic doses, which can be delivered endoluminally [5,10,94,97]. The addition of systemic chemotherapy may also improve tumour response, while local excision can be performed to treat small residual disease [43,98,99]. Multiple studies have explored the advantages and disadvantages of non-operative treatment modalities in selected groups of patients. Most of the performed studies reported on complete or near-complete response rates, rather than on local control. Nevertheless, the complete or near-complete response rates associated with a non-operative treatment modality may indicate its effect on the tumour response and the probability to obtain local control.

It has become clear that each modality may benefit each patient differently, supporting the need for a personalised treatment strategy [100]. Despite separate modalities as well as certain combinations having been explored, the optimal allocation in the elderly and frail is unknown. Centralisation of care to a dedicated centre with expertise on all non-operative treatment modalities in the elderly and frail seems warranted.

### 3.1. Systemic Chemotherapy

Adding systemic chemotherapy before or after (chemo)radiotherapy seems to improve local tumour response and may increase local control. Over recent years, the addition of systemic chemotherapy has been explored increasingly in studies on total neoadjuvant treatment [11,101]. Although some studies only reported small effects, promising response rates have been described in several randomised trials and cohort studies [102,103,104,105,106]. Calvo et al. reported significantly higher rates of tumour downstaging after adding systemic chemotherapy, which was also observed in a phase II study by Markovina et al. [107,108]. Meta-analyses by Petrelli et al. and Kasi et al. reported a pooled complete response rate of 22.4–29.9% in patients with locally advanced rectal cancer in whom systemic chemotherapy was added to (chemo)radiotherapy [98,109]. In patients with lower stages of rectal cancer, the complete response rates seem even higher. A study by Cercek et al. reported a complete response in 53.5% of patients with stage II disease [103]. However, the benefits of the addition of systemic chemotherapy for achieving local control and survival are unclear, especially in the elderly and frail. 

Systemic chemotherapy may induce toxicity, resulting in morbidity and decreased physical reserve capacity, particularly in the elderly and frail. The performed studies showed high compliance rates of 80–100% and similar toxicity rates when compared to chemoradiotherapy, but these studies were mostly conducted in relatively young and fit patients with a median age between 57 and 69 years [98,109]. Many studies investigated oxaliplatin-based chemotherapy, which is, particularly in the elderly, known for its adverse effects [110]. Studies exploring the effectiveness, the toxicity, and compliance of adding systemic chemotherapy in the elderly and frail are lacking. While it may be beneficial in relatively fit patients who refuse surgery, the absence of data and the potential toxicity probably limits its use in the non-operative management of the elderly and frail.

### 3.2. External Beam Radiotherapy (EBRT)

EBRT is most commonly administered in two different schedules: long-course chemoradiotherapy (45–50.4 Gy in fractions of 1.8–2.0 Gy with concomitant capecitabine) or short-course radiotherapy (SCRT) (25 Gy in fractions of 5 Gy).

Both schedules are associated with beneficial tumour response rates and form a viable basis for combinations in the non-operative management of rectal cancer. When compared to chemoradiotherapy, SCRT seems to result in slightly lower response rates. After chemoradiotherapy, a complete response is reported in 15–27% of patients with cT3–T4 rectal cancer [111,112]. Two population-based studies on data from the NCR showed that SCRT combined with a waiting interval of 4–5 weeks resulted in fewer complete (6.4–9.3% vs. 16.2–17.5%) and good (yT0–1) (11.0–17.5% vs. 20.6–22.6%) responses than chemoradiotherapy [113,114]. The Stockholm III trial reported significantly increased tumour regression rates in patients with a delayed interval (median 6.4 weeks) until surgery after SCRT, with a complete response in 11.4% of patients [115]. Response evaluation at 4–5 weeks after SCRT may be too early to evaluate the tumour response adequately. Furthermore, tumour response rates seem correlated with the initial tumour stage. In a pooled analyses by Maas et al. that included 3105 patients who underwent chemoradiotherapy, complete responses were observed in 58% of cT1, 28% of cT2, and 16% of cT3 tumours [112]. Most studies were performed in locally advanced rectal cancer and the response rates of chemoradiotherapy and SCRT in early stage tumours (cT1–3bN0) are relatively unexplored. The currently ongoing STAR-TREC phase II/III study (NCT02945566) is investigating the effects of chemoradiotherapy and SCRT on early stage rectal cancer and may provide valuable insights on the non-operative management of rectal cancer patients [116]. 

Earlier studies have shown that elderly patients treated with chemoradiotherapy achieved comparable response rates, disease-free survival, and tolerability in relation to their younger counterparts [6]. Data from the ACCORD12/PRODIGE2 phase 3 trial by François et al. reported that elderly patients treated with chemoradiotherapy had increased rates of grade 3 and 4 toxicity (25.6% vs. 15.8%) when compared to younger patients [117]. Still, 95.8% of the elderly successfully completed chemoradiotherapy [117]. While literature is controversial, SCRT seems associated with reduced toxicity. The preliminary results of the randomised NACRE study (NCT02551237) showed that all patients above 75 years old completed SCRT, while 14% did not complete chemoradiotherapy [118]. The number of serious adverse events (13 vs. 7 events) was also higher in patients treated with chemoradiotherapy [118]. A randomised trial by Bujko et al. reported less acute toxicity in patients treated with SCRT when compared to chemoradiotherapy (3.2% vs. 18.2%), while late toxicity was comparable (7.1% vs. 10.1%) [119]. Similar results were observed in a later meta-analysis [120].

When tolerated, chemoradiotherapy seems to be the most effective treatment for achieving local control in patients unable to undergo TME surgery [4,6]. SCRT has a shorter treatment duration and seems to result in lower toxicity, which may be preferable in frail or comorbid patients unfit for chemoradiotherapy or for whom treatment compliance might be a potential issue. 

Outcomes of other EBRT schedules (e.g., 13 × 3 Gy) on local control rates are scarce and unexplored. In the Lyon R90-01 trial, 29% of patients with cT2–T3 rectal cancer who were treated with 13 × 3 Gy EBRT achieved a complete or near-complete response after a waiting interval of 6–8 weeks [121]. These alternative schedules are currently under investigation, mostly in combination with dose escalating endoluminal radiotherapeutic boosts [42,122].

### 3.3. Dose Escalation of Radiotherapy

Radiotherapeutic dose-response analyses have showed that tumour responses can be improved by increasing the radiotherapy dose [94]. An earlier analysis by Appelt et al. showed that 72 Gy was needed to achieve a major tumour response in 50% of cT3–T4 rectal tumours [94]. Increased radiotherapy doses can be delivered by endoluminal radiotherapeutic modalities, such as contact X-ray brachytherapy (CXB) or high-dose rate endorectal brachytherapy (HDR-BT). Endoluminal radiotherapy has the ability to deliver high doses of radiotherapy directly to the tumour with a rapid dose fall-off, thus sparing normal surrounding tissue. If technically eligible, definitive dose escalations of radiotherapy are an attractive modality in elderly and frail patients unable to undergo TME surgery to maximise local control. These endoluminal interventions are only available in selected centres and should be surveilled by dedicated multidisciplinary teams.

#### 3.3.1. Contact X-ray Brachytherapy

The use of CXB is mainly described as a beneficial dose-escalating modality in patients unable to undergo surgery. Sun Myint et al. and Gérard et al. have described the use of CXB in rectal cancer patients as monotherapy (in early and small tumours), as an additional boost to EBRT, or as adjuvant treatment after local excision [97,123,124,125]. 

CXB as an additional boost to EBRT has been explored in multiple studies. In the Lyon R96-02 trial, a significant improvement in clinical complete response rates (24% vs. 2%) and pathological complete and near-complete response rates (57% vs. 34%) were observed in patients treated with an additional CXB boost after EBRT (13 × 3 Gy) when compared to EBRT (13 × 3 Gy) alone [122]. A multicentre phase II study by Gérard et al. showed that EBRT combined with a CXB boost resulted in complete and near-complete response rates of 95% in cT2–T3 rectal cancer [40]. Another study by the same group described complete and near-complete response rates after CXB and EBRT of 33–88% [126]. In a cohort described by Sun Myint et al., patients unsuitable for or refusing surgery achieved a complete response in 64–72%, of which 86–87% were sustained after a median follow-up of 2.5–2.7 years [39,123]. An additional 21–23% of patients with a clinical incomplete response had pathological complete responses after resection [39,123]. A recent study by Custers et al. reported on local control rates in older and inoperable rectal cancer patients who were treated with CXB after different schedules of radiotherapy (79%) or local excision (21%) [41]. The study showed that local control was achieved in 13 out of 19 (68.4%) patients, while 9 out of 19 (47.4%) patients had a clinical complete response [41]. The 1-year local progression-free survival was 78%, while the overall 1-year survival was 100% [41]. The quality of life was only slightly impaired and successfully returned to baseline after 6 months [41]. These results suggest that, if technically possible, CXB is an effective option in the elderly and frail to improve local control. Most studies were not randomised and did not include locally advanced tumours. The currently ongoing randomised OPERA trial (NCT02505750) and the OPAXX study will likely give more insights in the value of CXB in more advanced rectal tumours [127].

The reported toxicity rates of CXB are relatively low [39,123,128]. In the Lyon R96-02 trial, early and late grade III toxicity involved 9% and 11% of patients, respectively [40]. According to other studies, toxicity mostly included rectal bleeding. Grade I-III rectal bleeding occurred in 24–40% of patients, while grade III bleeding was described in <5% [39,125]. Rectal ulceration was described in 30% of patients, which was most often asymptomatic and usually healed within 3–6 months [39,125]. Functional outcomes after CXB are reported to be relatively good, with 65% of patients having no LARS complaints [40,129].

#### 3.3.2. High-Dose Rate Endorectal Brachytherapy

An alternative endoluminal dose-escalating modality to improve local control is HDR-BT [130]. Vuong et al. showed that a preoperative HDR-BT boost resulted in improved tumour response rates [131]. The study reported pT0N0-1 rates of 32%, while an additional 38% of patients only had small microscopic residual disease [131]. The beneficial effects of HDR-BT on the tumour response has been investigated in multiple other studies. In the phase I HERBERT study, 38 patients (median age of 83 years) were treated with 13 × 3 Gy EBRT followed by HDR-BT (3 fractions of 5–8 Gy) [42]. A clinical tumour response was observed in 29 out of 33 patients (87.9%), of which 20 patients achieved a complete response (60.6%) [42]. The 1-year local progression-free survival was 64% and the 1-year overall survival was 82% [42]. Overall grade 3/4 toxicity were observed in 33% and 4%, while acute and late grade 2/3 proctitis were observed in 81.6% and 88% of patients [42]. The authors concluded that HDR-BT provided good tumour responses, but had a considerable risk for toxicity in the elderly and frail. In a study by Garant et al., elderly patients (median age of 82 years) with mainly cT2–T3 tumours achieved a clinical complete response in 86.2% after 40 Gy of EBRT (in 16 fractions) followed by HDR-BT (3 fractions of 10 Gy) [132]. The 2-year local control rate was 71.5% [132]. In a randomised study by Jakobsen et al., cT3–T4 rectal cancer patients were treated with chemoradiotherapy followed by HDR-BT (2 fractions of 5 Gy), which resulted in a complete or near-complete response rate of 44% [133]. Toxicity mostly included diarrhea, skin problems and proctitis, but was comparable to those treated with chemoradiotherapy alone [133]. Appelt et al. described that 40 out of 51 (78.4%) patients with cT1–3ab rectal cancer achieved a complete response after chemoradiotherapy followed by a 5 Gy boost of HDR-BT, with 2-year local control rates of 58% [134]. These patients had relatively good functional outcomes, as 69% of patients did not report faecal incontinence [134]. Based on these results, HDR-BT may be a useful modality to improve tumour response and optimise local control. Currently, the randomised HERBERT-II study (Netherlands Trial Register: NL7795) is investigating the additional effect of HDR-BT (3 × 7 Gy) after EBRT (13 × 3 Gy) in elderly and frail patients unable to undergo surgery.

### 3.4. Local Excision

Early rectal cancer can be treated with local excision with relatively low risks for morbidity and mortality, and relatively good functional outcomes [4,135]. Over the years, the indication for local excision has been broadened. However, long-term results reported local recurrence rates after a primary local excision of pT2 tumours up to 37% [136]. In the CARTS-study, patients with cT1-3N0 rectal cancer underwent chemoradiotherapy followed by local excision in case of residual ycT0-2N0 disease [43]. The study reported successful organ-preservation in 64% of patients with residual ycT0-2N0 disease, and in 55% of all patients that started with chemoradiotherapy [43]. A meta-analysis that investigated chemoradiotherapy followed by local excision in cT2–T3 rectal cancer showed adequate local control rates, with no recurrences in patients with a pathological complete response, while the recurrence rates were 2%, 7%, and 12% in patients with ypT1, ypT2, and ypT3 disease, respectively [137]. Additionally, several other studies reported comparable local control rates after local excision in patients after chemoradiotherapy [137,138,139]. However, local excision preceded by neoadjuvant treatment seems to result in an increased risk for wound infections, wound dehiscence and severe functional bowel complaints [138,140,141]. In the long-term follow-up of the CARTS-study, major LARS was observed in 50% of patients [140]. Local excision may be reasonable to treat small residual disease after chemoradiotherapy or SCRT in the elderly and frail unable to undergo completion TME surgery [142]. Nevertheless, selecting the patients that benefit most seems challenging. The outcomes in locally advanced rectal cancer are unknown. More insights will probably be gained by the currently ongoing OPAXX study, which randomises patients with more advanced rectal cancer and a near complete response between CXB and the extension of the waiting interval followed by a local excision [127].

## 4. Response Evaluation and Follow-Up

### 4.1. Response Evaluation

Since the primary aim in treating elderly and frail rectal cancer patients unable to undergo TME surgery is to achieve local control of disease, response evaluation could be relevant to consider additional local treatment options, such as endoluminal radiotherapy or local excision. Earlier studies by Maas et al., showed that digital rectal examination, magnetic resonance imaging (MRI) with diffusion-weighted imaging (DWI), and endoscopy is the most accurate combination to evaluate tumour response [143]. MRI-DWI is a suitable imaging technique to identify good responders, with high positive predictive values up to 91% for tumour downstaging and downsizing confined to the rectal wall [144]. MRI-DWI seems to be able to identify potential candidates for additional endoluminal radiotherapy or local excision. In these patients, an endoscopy performed by a dedicated gastroenterologist can further characterise the tumour. The endoscopy is preferably performed in the presence of a dedicated surgical oncologist and radiation oncologist to identify the eligibility for local treatment options.

The current standard between the end of radiotherapy and response evaluation is 6–8 weeks, which is also considered as a beneficial period to select patients for additional local treatment options. It has been shown that the tumour response increases over time. Earlier studies have reported ongoing tumour responses up to 22–26 weeks after finishing EBRT [145,146,147]. In some cases, it might, consequently, be necessary to lengthen the interval before considering additional local treatment modalities to allow further tumour shrinkage. In these patients, a re-assessment of the tumour response at a later interval may be relevant.

In elderly and frail patients who are unable to undergo TME surgery, response evaluations should not be performed routinely, but should be adapted to the individual patient, treatment goals, and the relevance for considering additional treatment options.

### 4.2. Follow-Up

The follow-up of elderly and frail patients unable to undergo TME surgery should be tailored and patient-centred. The follow-up will probably have different goals than clinicians are used to in the follow-up of young and fit patients. Especially in those in whom relapsing disease would have no clinical consequences, the oncological benefits of the follow-up are negligible. However, the follow-up in elderly and frail patients should be considered as an important period to monitor the patient’s health status and prevent functional decline. Clinicians should realise that, although the treatment might have finished, the care for these patients never stops. 

Many patients experience a decline in their level of independence after intensive treatment [27,28,29,30]. Yet, while most patients are able to return to baseline levels within 3–6 months, they face a long-term risk for functional decline [27,28,29,30]. The follow-up in these patients should aim at preventing functional decline and loss of independence, and at monitoring the consequences of treatment or tumour progression. A personalised follow-up plan is required, considering the benefits of the early detection of relapsing disease or functional decline, versus the burden of follow-up.

Apart from the treating physician, the general practitioner has a crucial role during the follow-up of these patients. A study showed that most patients preferred contacting the general practitioner to discuss problems related to nutrition, physical condition, and fatigue, which are common contributors to the onset and progression of functional decline [148]. On the other hand, patients were inclined to discuss disease-specific complaints with their treating physician [148]. Hence, effective communication and alignment of care between the treating physician and the general practitioner is required. This has been supported by a Dutch study among 140 older colorectal cancer patients (≥70 years). The study showed that a standardised transmission of communication between the treating physician and the general practitioner, combined with regular follow-up by the general practitioner resulted in improved health, reduced frailty, and increased quality of life [149]. This supports that elderly and frail patients require close monitoring after treatment to detect early signs of functional decline. Promising shared-care models between the general practitioner, the geriatrician, and the treating physician have been described to improve outcomes during and after treatment [150,151]. 

A geriatric assessment is not only valuable to personalise treatment, but also to tailor follow-up with targeted interventions [53]. In a randomised study, in which elderly patients (mean age 82.5 years) underwent follow-up based on a geriatric assessment, reduced 3-year mortality rates and higher patient satisfaction were observed [152]. Rehabilitation programmes improve treatment-induced functional decline and long-term side effects caused by chemotherapy and radiotherapy (e.g., fatigue, reduced physical condition, weight changes and cognitive deterioration), resulting in improved quality of life [153,154,155]. This may help patients to improve their health status, return to their baseline level of functioning, and prevent functional decline.

## 5. Multidisciplinary Clinical Care Pathway

### 5.1. A Practical Suggestion of a Multidisciplinary Care Pathway

Based on a literature review, the authors of this study have successfully implemented a multidisciplinary care pathway in the Catharina Hospital (Eindhoven, The Netherlands) for elderly and frail rectal cancer patients unable to undergo TME surgery. The clinical care pathway was developed in close collaboration with an advisory board of rectal cancer patients to improve personalised care within the multidisciplinary clinical care pathway.

All rectal cancer patients considered frail by the treating physician enter the clinical care pathway. First of all, the patient is discussed and the diagnostic work-up is evaluated by the multidisciplinary team. The expertise of the multidisciplinary team treating these patients is ensured by the attendance of a dedicated surgical oncologist, medical oncologist, radiation oncologist, and a geriatrician, all with special interest on the non-operative management of elderly and frail rectal cancer patients. 

After the multidisciplinary team meeting, the patient is admitted in day care and evaluated by the members of the multidisciplinary team. In some cases, the anaesthesiologist is also part of the multidisciplinary team to assess the tolerability of surgery and anaesthesia, if applicable. After each individual physician has assessed and informed the patient about the benefits and risks of the treatment options, a second multidisciplinary team meeting is organised. The patient’s health status, preferences, and treatment goals are discussed by the concerning physicians. The outcomes of the comprehensive geriatric assessment have a central role within the multidisciplinary discussion. The multidisciplinary team conscientiously composes a personalised non-operative treatment advice, aiming to provide local control of disease, prevent the onset of debilitating symptoms, prolong survival, and maintain functional independence and quality of life. The personalised treatment advice may vary from an intensive trajectory of systemic therapy, followed by chemoradiotherapy, and endoluminal brachytherapy in fairly fit patients who refuse surgery, to short-course radiotherapy schedules or no treatment at all in the frailest patients. The treatment advice, including the benefits and risks, is communicated with the patient and their relatives, who are actively involved in the decision-making process. After the treating physician and the patient have weighed the benefits and risks, a definitive treatment plan is proposed. 

**Figure 1:**
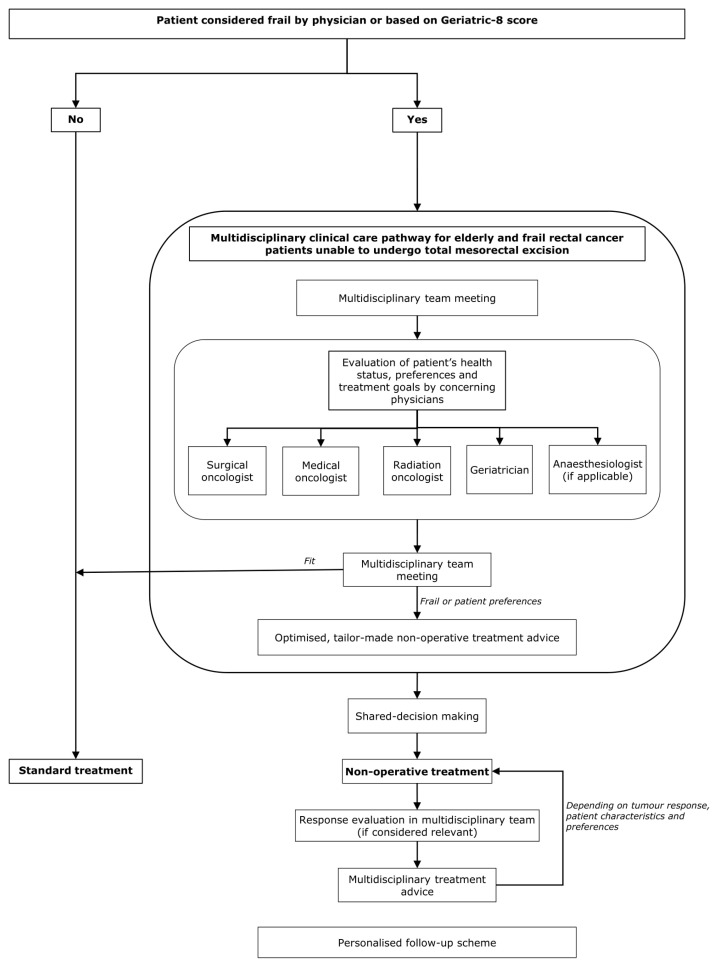
Flowchart of the multidisciplinary clinical care pathway for elderly and frail rectal cancer patients that has been successfully implemented by the authors of the study.

After treatment has finished, response evaluation and follow-up are adapted to the individual patient, treatment goals, and the relevance for considering additional treatment options. Figure 1 presents a flowchart of the multidisciplinary clinical care pathway.

### 5.2. Future Perspectives

Limited knowledge is available on the outcomes of the non-operative management of elderly and frail rectal cancer patients. The lack of data impedes counselling patients with a doubtful health status or unwillingness to undergo TME surgery. Clinicians are unable to provide adequate information about short-term and long-term outcomes. Prospective studies evaluating the treatment and outcomes of elderly and frail patients unable to undergo TME surgery are warranted to improve the decision-making process.

The authors of the present study have initiated a currently ongoing single-centre prospective observational cohort study, named the RESORT study (A Prospective Registry of the Non-Invasive Multimodality Treatment in Inoperable Rectal Cancer Patients: Evaluating the Current Treatment Strategies in Rectal Cancer Patients Unable to Undergo TME Surgery). The aim of the study is to evaluate the decision-making process, the treatment, and the outcomes of elderly and frail rectal cancer patients unable to undergo TME surgery. Patients unable to undergo TME surgery and treated in the multidisciplinary clinical care pathway in the Catharina Hospital are eligible for inclusion. After informed consent is obtained, the study prospectively collects data during a follow-up of 3 years. The data collection includes information regarding patient characteristics, diagnosis, treatment, local control of disease, survival, quality of life, and functional outcomes (Table 2).

## 6. Conclusions

In elderly and frail rectal cancer patients unable to undergo TME surgery, non-operative treatment strategies may offer a viable alternative, aiming to obtain local control of the primary tumour. Elderly and frail patients often prioritise quality of life and maintaining independence over oncological outcomes. The challenge in treating the elderly and frail is to maximise the effectiveness of treatment by optimising the balance between the probability for maximal local control, the patient’s preferences, and the burden of treatment. Personalisation of care is of utmost importance and requires a patient-centred approach, in which the patient is actively involved. A comprehensive geriatric assessment is considered as a crucial element and should have a central role in the multidisciplinary discussion. Response evaluation and follow-up should be adapted to the individual patient, treatment goals, and clinical relevance as well. 

Although an increasing number of elderly and frail patients are treated non-operatively, limited knowledge is available on the optimal non-operative management of this patient group. Elderly and frail patients unable to undergo TME surgery should be treated in specific clinical care pathways by dedicated multidisciplinary teams with expertise on the non-operative management of these patients. Based on the literature review, we provided a practical suggestion of a successfully implemented clinical care pathway, in which patients are assessed and discussed multidisciplinary to personalise treatment. Future studies regarding the treatment and outcomes of the elderly and frail unable to undergo TME surgery are needed to improve decision making. The authors of this study have initiated a currently ongoing prospective observational cohort study (RESORT study) to investigate the outcomes of a multimodal and patient-centred non-operative treatment approach for elderly and frail rectal cancer patients unable to undergo TME surgery. The study will provide important data regarding the decision-making process, treatment, and outcomes.

This narrative review provides a robust literature review and a practical suggestion for a multidisciplinary clinical care pathway in order to assist rectal cancer experts in improving and personalising the care for elderly and frail rectal cancer patients.

## Figures and Tables

**Table 1:** Elements of the comprehensive geriatric assessment within the non-operative management of elderly and frail rectal cancer patients.

Geriatric Domain	Examples of Scoring Tools
Age	-
Functional status	Eastern Cooperative Oncology Group (ECOG) Performance status [65] Karnofsky Performance status [66]
Level of independence	Katz scale of Activities of Daily Living (ADL) [67] Lawton and Brody scale of Instrumental Activities of Daily Living (IADL) [68]
Comorbidity	Charlson Comorbidity Index (CCI) [69]
Medication use	Number and type of medication use
Physical function and mobility	4-Meter Gait Speed [70] Handgrip strength [71] Timed Up and Go (TUG) [72]
Cognitive function	Six-item Cognitive Impairment Test (6-CIT) [73] Mini-Cog [74] Visual Association Test (VAT) [75] Clock Drawing Test (CDT) [76] Mini Mental State Examination (MMSE) [77]
Emotional function	Patient Health Questionnaire (PHQ-2) [78] Geriatric Depression Scale-15 (GDS-15) [79] Hospital Anxiety and Depression Scale (HADS) [80]
Nutritional status	Mini-Nutritional Assessment Short Form (MNA-SF) [81] Body Mass Index (BMI)
Social status	Living arrangements (independent, institutionalised, hospitalised) Availability of an informal and formal caregivers (number of days with home care)
Geriatric risk factors or syndromes	Risk to fall/fall history Risk of delirium Vision or hearing difficulties Pain Urinary and/or faecal incontinence
Treatment goals and preferences	e.g., Minimising/improving local complaints related to the tumour Maintaining/improving quality of life Maintaining/improving functional status Prolonging survival

**Table 2:** Overview of the variables for data collection in the RESORT-study.

Variable Group	Variables	
Patient characteristics	Age Sex Weight, length, BMI Living situation	Medical history ECOG Performance status Comorbidities
Primary diagnosis	Clinical complaints Tumour location Tumour size	TNM stage Histology
Geriatric assessment	Geriatric scoring tools (e.g., Katz-ADL, Lawton and Brody-IADL, MNA-SF, 6-CIT, Mini-Cog, 4-Meter Gait Speed) Clinical Frailty Score Treatment goals and preferences	
Multidisciplinary evaluation	Considerations of the multidisciplinary team	Treatment advice
Treatment	Treatment modalities Treatment schedules	Compliance rates Adverse effects/complications
Response evaluation	Tumour response Tumour characteristics	Multidisciplinary advice on response evaluation
Follow-up	Clinical complaints Local control rates Relapsing disease (local/distant)	Survival outcomes Date of death (if applicable) Cause of death (if applicable)
Quality of life and functional outcomes ^2^	EORTC ^1^ QLQ-C30 EORTC QLQ-CR29 EQ-5D-5L	Katz-ADL Lawton and Brody IADL

^1^ EORTC, European Organisation for Research and Treatment of Cancer. ^2^ At baseline and after 6 months, 12 months, 24 months, and 36 months after finishing treatment.
